# Autologous Enhancement by Interferon of Natural Killer Activity of Human Peripheral Blood Lymphocytes

**DOI:** 10.1155/S0962935194000475

**Published:** 1994

**Authors:** S. B. Cheknev, Y. G. Ashmanova, A. D. Pritsker, O. L. Latysheva, F. I. Yershov, A. J. Kulberg

**Affiliations:** Laboratory of Immunochemistry and Department of Interferons N. F. Gamaleya Institute for Epidemiology and Microbiology of the Russian Academy of Medical Sciences Moscow 123098 Russia

## Abstract

The *in vitro* action of interferon (IFN)-α and IFN-γ from six healthy donors and ten patients with multiple sclerosis (MS) on natural killer (INK) activity of peripheral blood lymphocytes (PBL) was studied in an autologous system. The NK activity of PBL was detected by a cytotoxic test using ^3^H-uridine human erythromyeloblast K562 cells. Autologous IFN-α and IFN-γ did not augment NK activity of PBL from healthy donors *in vitro*, whereas in samples from MS patients the IFNs strongly stimulated NK cell cytotoxic function. This stimulation suggests the existence of an inhibitor of regulatory IFN action, that is produced in healthy donors simultaneously with IFN in response to IFN induction, but which is lacking in commercial IFN preparations. The factor-containing supernatants from healthy donors reduced the stimulatory action of autologous IFNs in patients with MS almost until complete blockade. Because this inhibitor was absent in patients with MS, deficiency of an inhibitor of IFN regulatory action in MS could open the way to treatment of this compartment of the immune system.


Mediators of Inflammation 3, 341–346 (1994)

THE *in vitro* action of interferon (IFN)-α and IFN-γ from six healthy donors and ten patients with multiple sclerosis (MS) on natural killer (NK) activity of peripheral blood lymphocytes (PBL) was studied in an autologous system. The NK activity of PBL was detected by a cytotoxic test using $^3$H-uridine human erythromyeloblast K562 cells. Autologous IFN-α and IFN-γ did not augment NK activity of PBL from healthy donors *in vitro*, whereas in samples from MS patients the IFNs strongly stimulated NK cell cytotoxic function. This stimulation suggests the existence of an inhibitor of regulatory IFN action, that is produced in healthy donors simultaneously with IFN in response to IFN induction, but which is lacking in commercial IFN preparations. The factor-containing supernatants from healthy donors reduced the stimulatory action of autologous IFNs in patients with MS almost until complete blockade. Because this inhibitor was absent in patients with MS, deficiency of an inhibitor of IFN regulatory action in MS could open the way to treatment of this compartment of the immune system.

**Key words:** Autologous interferon, Enhancement, Human peripheral blood lymphocytes, Natural killer activity

### Autologous enhancement by interferon of natural killer activity of human peripheral blood lymphocytes

**S. B. Cheknev,[CA] Y. G. Ashmanova, A. D. Pritsker, O. L. Latysheva, F. I. Yershov and A. J. Kulberg**

Laboratory of Immunochemistry and Department of Interferons, N. F. Gamaleya Institute for Epidemiology and Microbiology of the Russian Academy of Medical Sciences, Moscow 123098, Russia

[CA] Corresponding Author

### Introduction

The ability of interferons (IFNs) to activate natural killer (NK) cells is an important property of these mediators.[1] By inducing differentiation of pre-NK to active NK forms, enhancing the lytic potency of a single killer cell, and accelerating the recycling of NK cells after lytic interaction with their targets[2,3] IFNs serve factors playing a key role in the function of this lymphocyte population.[1,2]

The spectrum of immunomodulatory action of IFNs, as well as their strong antiproliferative activity,[4] has led to the use of IFN preparations in the therapy of oncological diseases.[5,6] However, in spite of proven clinical efficiency, IFN action does not correspond to expectation in patients with idiopathic autoimmune haemolytic anaemia *in vitro*,[7] in a course of IFN therapy in chronic granulocytic leukaemia[8] or in myelodisplasia patients.[9]

Multiple sclerosis (MS) is a disorder where the effects of IFN are puzzling.[10–12] If the use of IFN-α and IFN-β is characterized by a reduction in the number and severity of exacerbations[11,12] then IFN-γ treatment leads, at least in some patients, to exacerbation of the pathological process.[11,13] MS is associated not only with a strong deficiency of cytotoxic NK cell activity[14–17] and a functional defect of the IFN system,[15,16,18,19] but also anomalous responses of NK cells to regulatory IFN action.[14,17,20]

NK cells produce multiple cytokines including IFN,[21] and the reduced IFN production by peripheral blood lymphocytes (PBL) possessing NK activity,[15] in patients with MS, is probably related to a defect in monocyte helper.[22] There also appears to be a dissociation between the cytotoxic and IFN-producing activities of PBL exhibiting NK properties in this disease,[23] as well as maintenance of an excess of IFN in the serum of blood and cerebrospinal fluid in these patients.[19,24] In the light of these relationships, the aim of the present work was to study the autologous (auto-) IFN-α and IFN-γ actions on the cytotoxic NK activity of PBL in patients with MS and in healthy donors.

### Materials and Methods

Six healthy donors (two men and four women) aged between 18 and 48 years, and ten patients (four men and six women) aged between 18 and 42 years with evident MS[25] with remitting course of the process at the stage of exacerbation, cerebrospinal form and various duration of the disease (from 3 to 8 years), and IV–V degrees of invalidization (Kourtske scale) were investigated.

Determination of the action of auto-IFN on NK activity of PBL was carried out in two steps. Initially, peripheral venous blood of patients was collected for IFN induction while PBL were used to evaluate NK cytotoxicity. Then, after IFN induction (on the 8th day of investigation), PBL were isolated from peripheral blood of the same healthy donors and patients with MS (MS patients received a placebo trial), and the action of auto-IFN on NK activity of these PBL was determined.

© 1994 Rapid Communications of Oxford Ltd

PBL were isolated from patients' blood on Ficoll–Paque (Pharmacia Fine Chemicals) gradients of a density of 1.077 g/cm$^3$ using the method of Böyum.[26]

The cytotoxic NK activity of PBL was measured by the radiometric assay of Hashimoto and Sudo,[27] as modified by Rykova *et al.*,[28] using target cells of the standard human erythromyeloblast line K562 labelled with $^3$H-uridine at a dose of 3 µCi/ml. PBL and K562 cells were inoculated into the U-shape wells of 96-well microplates followed by co-incubation for 14 h at 37°C in a humid atmosphere containing 5% $CO_2$. The incubation was carried out in 0.2 ml RPMI-1640 medium (Amimed) together with 12% v/v foetal calf serum (NF Gamaleya Institute for Epidemiology and Microbiology, Moscow), 10 mM HEPES (Serva), 2 mM glutamine, and 40 µg/ml gentamicin (Pharmachim). NK activity was tested over a wide range of effector:target cell (E:T) ratios — 100:1, 50:1, 25:1, 12:1 and 6:1. Each E:T ratio was prepared in two or three parallel wells. Initial suspensions of PBL contained $10^7$ PBL/ml, and the suspension of K562 targets contained $10^5$ cells/ml. After stopping co-incubation the PBL and K562 cells were harvested by a 12-channel harvester (Dynatech) on glass fibre filters with 2.5 µm pores (Whatman). Radioactivity remaining on the filters was counted for 1 min for each probe using β-counters (Packard Mark II, USA).

The index of cytotoxicity (IC) was determined according to the formula:

$$IC = \left(1 - \frac{\text{cpm in testing well}}{\text{cpm in control well}}\right) \times 100\%$$

Control wells contained K562 cells which were labelled, prepared and incubated in the same manner as those of test wells, but without PBL.

IFN-α and IFN-γ were induced in samples of whole blood by Newcastle Disease Virus (Kansas strain) at the dose of 1 CPU/ml and by phytohaemagglutinin P (Difco) at the dose of 5 µg/ml over 24 h at 37°C in an atmosphere of 5% $CO_2$ using the method of Kirchner *et al.*,[29] as modified by Grigoryan *et al.*[30] Inactivation of the inducing virus in IFN-α-containing supernatants was achieved by adding 1N HCl to reduce the pH to 2.0. The mixture was incubated for 72 h at 4°C then neutralized by adding 1N NaOH to give a pH of 7.0. Titration of IFN was provided in M19 monolayer cultures of human fibroblasts according to the method of Solovev and Bektemirov.[31] Vesicular stomatitis virus (VSV) of Indiana strain was used as a test virus. The reverse dilution of IFN which was sufficient for a 50% protective effect of the IFN on cells infected with 100 $CPD_{50}$ was regarded as 1 unit of the IFN titre.

To study IFN action on NK activity of PBL, 0.1 ml of the IFN-containing supernatant was added to PBL suspension ($10^7$ PBL/ml in 0.4 ml of the medium) for 1 h at 37°C, then PBL washed free of IFN were inoculated into wells of microplates. Because of the lack of results for the induced IFN titration until the second step of the investigation, the dosage of IFN was controlled by adjusting the volume of added supernatant.

Efficiency of IFN action was determined by regulatory parameters (RPs) presenting the ratios of absolute changes of the IC to initial NK activities, counted as mean values for all tested E:T ratios and expressed with corresponding signs.[20] Total RP (TRP) for a group of observations was counted as mean value of RPs in this group. Student's *t*-test was used for evaluation of the statistical significance of the results.

### Results

The data in Fig. 1A show that auto-IFNs did not augment the cytotoxic NK activity of PBL obtained from blood of healthy donors. The TRP of auto-IFN-α was equal to $-8 \pm 16\%$ and of auto-IFN-γ to $6 \pm 10\%$. At the same time auto-IFNs strongly activated PBL possessing NK activity in patients with MS (Fig. 1B). The TRPs were $28 \pm 11\%$ ($p < 0.05$) for auto-IFN-α and $56 \pm 26\%$ ($p < 0.05$) for auto-IFN-γ.

Comparison of the doses of auto-IFNs in the study of IFN action on NK activity of PBL shows that the same doses of auto-IFNs were used in normal individuals as in MS patients (Table 1). Moreover, these doses of auto-IFNs were similar to those adopted in previous work concerning the regulatory action of commercial IFN preparations on PBL NK cytotoxicity,[20] where the difference was not more than one dilution of IFN-containing supernatant.

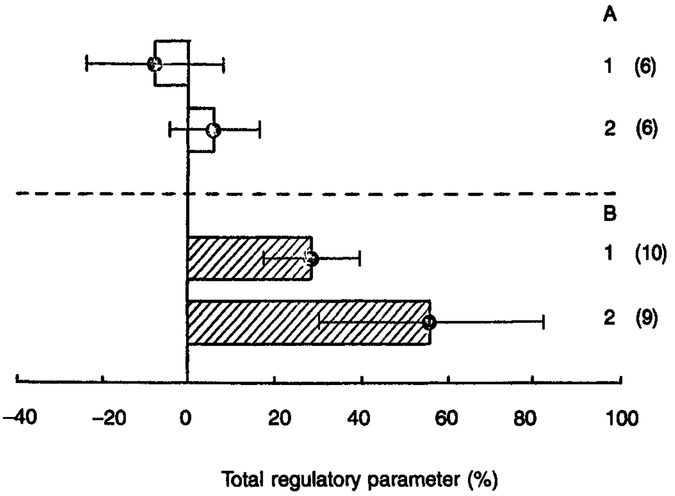

Fig. 1. Action of auto-IFN-α (1) and auto-IFN-γ (2) *in vitro* on human NK activity of PBL in healthy donors (A) and patients with MS (B). $10^7$ PBL/ml in a volume of 0.4 ml were treated with auto-IFN-α or auto-IFN-γ for 1 h at 37°C followed by washing free of IFNs and co-incubation with K562 targets for 14 h at 37°C in humid atmosphere containing 5% $CO_2$. Shading: $p < 0.05$ compared with TRP in healthy donors. The number of observations is displayed in parentheses. One observation corresponds to RP counted for one subject's probe of PBL.

**Table 1.** Doses of auto-IFNs used in the study of IFN action on PBL NK activity

| Donors | Type of auto-IFN | Dose of IFN (IU/ml)* | Dose range (IU/ml) |
|---|---|---|---|
| Healthy donors | IFN-α ($n=6$) | 136.8 ± 19.8 | 51.2 – 209.6 |
| | IFN-γ ($n=6$) | 32.2 ± 8.4 | 12.8 – 112.2 |
| MS patients | IFN-α ($n=10$) | 164.2 ± 24.1 | 46.8 – 409.6 |
| | IFN-γ ($n=9$) | 31.4 ± 6.2 | 1.6 – 103.4 |

*Concentration of auto-IFNs in final PBL suspension is displayed. Because of the lack of results for the auto-IFN titration until the second step of the investigation, the dose of IFN was controlled by adjusting the volume of supernatant added to PBL suspensions.

Thus, the described difference in properties of auto-IFNs that stimulate NK activity of PBL in MS patients, compared with those in healthy donors, was not related to the other IFN doses used in the present study.

The distribution of regulatory influences of auto-IFNs on PBL NK activity was compared in MS patients and in healthy donors. As can be seen from Table 2, the number of observations when stimulation or inhibition of NK activity of PBL, or absence of an effect were seen, using all E:T ratios, were similar in normal individuals and MS patients. Further, the number of observations in which auto-IFNs did not augment PBL NK cytotoxicity was equal to 68% for IFN–α and 67% for IFN–γ in healthy donors, and 67% for IFN–α and 63% for IFN–γ in MS patients (Table 2).

**Table 2.** Distribution of the *in vitro* effects of auto-IFNs on human PBL NK activity (number of observations)*

| Donors | Type of IFN | Stimulation of NK activity | Absence of effects | Inhibition of NK activity |
|---|---|---|---|---|
| Healthy donors | IFN-α | 8 (32)** | 11 (44) | 6 (24) |
| | IFN-γ | 10 (39) | 10 (39) | 6 (22) |
| MS patients | IFN-α | 13 (33) | 14 (36) | 12 (31) |
| | IFN-γ | 11 (37) | 11 (37) | 8 (26) |

*One observation corresponds to the value of IC determined for one definite E:T ratio in one subject. The summarized data for all tested E:T ratios are presented.

**Number of observations in per cent is displayed in parentheses.

The modulatory action of auto-IFNs on PBL possessing NK cytotoxicity in MS patients was the same as in healthy donors (Table 3), and these data may indicate that the stimulatory effects of auto-IFNs detected in MS patients were not related to changes in the properties of these IFNs themselves or in the sensitivity of the cells to regulatory IFN influence, thus suggesting the existence and special role of some additional factor controlling the regulatory properties of IFNs.

Evaluation of NK activity of the blood cells obtained from healthy donors and MS patients on the 1st and 8th days of study showed that NK activity of PBL, expressed in both mean values of the IC and comparison of the spectrum of its variability, did not change over 1 week (Table 4). Thus, a change in NK activity of PBL in analysing IFN regulatory action on the cytotoxic function of the cells can be excluded. Moreover, the data in the Table 4 show that PBL NK activity in MS patients was lower than in healthy donors but was sufficient for the evaluation of IFN action, whether enhancing or reducing.

A possible influence of PHA, which may be present in IFN–γ-containing supernatants, could be also excluded. Control tests were performed, including a study of *in vitro* PHA P action on PBL NK activity in three healthy subjects and four MS patients. PHA P was used at doses equal to those inducing IFN–γ or auto-IFN-γ production. PHA P did not affect *in vitro* PBL NK cytotoxicity in either healthy donors or MS patients (data not shown).

The IFN-containing supernatants obtained from PBL of healthy donors, acting on IFN-enhanced NK activity of MS patients' PBL completely abolished the stimulatory effect of auto-IFNs on PBL NK cytotoxicity in MS patients (Fig. 2). The action of the suggested inhibitor of IFN regulatory action on NK

**Table 3.** The relationship between initial NK cytotoxicity and the effects of auto-IFNs on PBL NK activity. Results are given for MS patients and healthy donors

| Initial NK activity of PBL | | IFN-α | | | IFN-γ | | |
|---|---|---|---|---|---|---|---|
| | | Stimulation | Absence of an effect | Inhibition | Stimulation | Absence of an effect | Inhibition |
| **Healthy donors** | | | | | | | |
| High | $n=3$ | 1 | – | 2 | 1 | 1 | 1 |
| Normal | $n=2$ | 1 | 1 | – | 2 | – | – |
| Low | $n=1$ | 1 | – | – | 1 | – | – |
| **MS Patients** | | | | | | | |
| High | $n=3$ | 1 | – | 2 | – | 1 | 2 |
| Normal | $n=1$ | – | 1 | – | – | 1 | – |
| Low | $n=6$ | 3 | 2 | 1 | 5 | 1 | – |

($n$ is the number of observations)

**Table 4.** Dynamics of NK activity of PBL on the 1st and 8th days of the study (IC in per cent, M ± m)

| | E:T ratio | | | | |
|---|---|---|---|---|---|
| | 100:1 | 50:1 | 25:1 | 12:1 | 6:1 |
| **Healthy donors** ($n = 6$) | | | | | |
| 1st day of study | 79.3 ± 4.7 (70–82)* | 64.2 ± 8.0 (48–81) | 61.4 ± 3.2 (54–68) | 56.8 ± 7.1 (47–76) | 52.1 ± 10.4 (41–78) |
| 8th day of study | 79.3 ± 9.1 (73–88) | 64.7 ± 5.9 (53–77) | 60.3 ± 5.7 (49–73) | 54.2 ± 5.2 (36–74) | 50.1 ± 15.0 (33–85) |
| **MS patients** ($n = 10$) | | | | | |
| 1st day of study | 58.1 ± 5.0 (22–74) | 67.2 ± 3.4 (57–76) | 40.0 ± 7.2 (5–78) | 33.3 ± 3.8 (16–54) | 29.7 ± 8.1 (4–50) |
| 8th day of study (after 1 week placebo trial) | 52.3 ± 8.8 (16–74) | 54.8 ± 8.7 (12–84) | 52.0 ± 6.1 (18–87) | 32.4 ± 7.7 (16–61) | 27.1 ± 6.0 (8–44) |

*Range of the IC values is displayed in parentheses.

activity was evaluated by adding it, at doses corresponding to those used for induced auto-IFNs, to a PBL suspension stimulated with auto-IFN, and incubating the mixture as described above for auto-IFNs. After this procedure TRPs became equal to 6 ± 5% ($p < 0.05$) for IFN-α and to −9 ± 7% ($p < 0.05$) for IFN-γ. The difference was statistically significant for both these auto-IFNs (Fig. 2), and the values of TRPs corresponded to those obtained for PBL from MS patients possessing NK activity without any treatment. This abolition of the IFN stimulatory effect on PBL NK cytotoxicity by allogeneic factors was not surprising because a negative regulation was found to be efficient not only for autologous but also for allogeneic systems in healthy donors (data not shown).

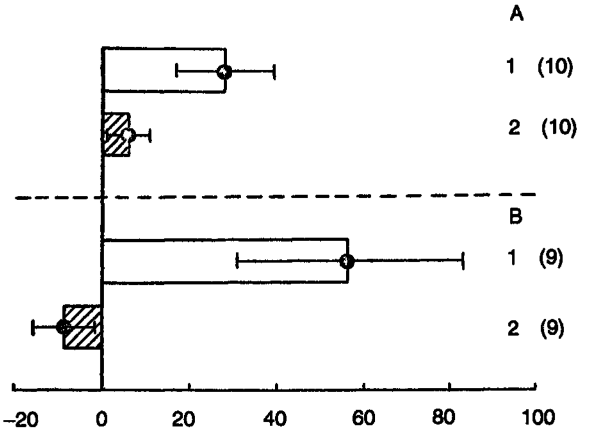

Fig. 2. Blockade of stimulatory actions of auto-IFN-α (A) and auto-IFN-γ (B) on NK activity of MS patients' PBL by IFN-containing supernatants obtained from PBL of healthy donors. The probes of IFN-containing supernatants obtained from PBL of healthy donors in a volume of 0.1 ml were added to PBL suspensions of MS patients simultaneously with 0.1 ml of auto-IFN-containing probes for 1 h at 37°C followed by washing free of preparations and measurement of PBL NK cytotoxicity (2). Shading: $p < 0.05$ compared with TRP of auto-IFNs alone (1). The number of observations is displayed in parentheses. One observation corresponds to RP counted for one subject's probe of PBL.

### Discussion

It has been shown in this study that non-purified auto-IFN-α and auto-IFN-γ did not stimulate NK activity of PBL in healthy donors. This is a surprising observation and contradicts those of Saksela *et al.*[2] and Targan and Dorey.[3] However, in these earlier studies a partially purified human leukocyte IFN was used[2] and a commercial preparation of human leukocyte IFN was applied,[3] whereas we worked with IFN preparations that had not been purified. Additionally, we showed in earlier investigations that commercial preparations of human leukocyte IFN, as well as IFN-γ at doses of 50 U/ml and 125 U/ml, stimulated NK cytotoxic activity of PBL, with TRPs equal to 270 ± 106% and 143 ± 61%, respectively, for IFN-α; and to a lesser extent, but also significantly, with TRPs of 46 ± 16% and 31 ± 7.5%, respectively, for IFN-γ.[20] The doses of IFNs used in this study were similar to some used by Saksela *et al.*[2]

At the same time as auto-IFN-α, auto-IFN-γ strongly augmented NK activity of PBL obtained from blood of MS patients. Results from the control experiments showed no change in either the immunomodulatory properties of IFNs on PBL NK activity, or the state of the PBL population possessing NK activity. Additionally, in MS patients, there was no change in the sensitivity of cells to the regulatory effect of IFN, compared with similar effects in healthy donors. Thus the present study seems to show the first evidence of an inhibitor of IFN regulatory action (IIRA) induced by IFN inducers and released simultaneously with IFN-α and, more importantly, with IFN-γ. The nature of the IIRA remains unknown, but as its production is associated with IFN induction, and its blocking efficiency depends on the initial NK activity of PBL, this factor might be one of a family of IFN inhibitors described in previous studies.[32,33] The lack of IIRA in commercial IFN preparations adds further weight to this suggestion. However, unlike the Fleishmann *et al.*,[32] or the Marchenko *et al.*,[33] factors, which abolished antiviral IFN effects, the properties of our IIRA are expressed through a reduction in the efficiency of IFN-α and IFN-γ stimulatory influences on PBL possessing NK activity. On the other hand, an inhibitor of the antiviral IFN action contained in the ultrafiltrate of the culture fluid obtained by induction of IFN on MCB murine cells with NDV was concentrated after neutralization of NDV in thin-channel wells of the TCF-10 apparatus, and abolished the regulatory effect of IFN on the sensitivity of murine L929 targets to a human NK cell-mediated cytolysis.[34] At the same time, Cheknev *et al.*[34] also showed that incubation of the IFN-resistant target cells of the MCB cell line led to an effect similar to the well-known protective action of IFN itself. The data obtained may therefore serve as a confirmation of the existence of a family of IFN inhibitors, a part of which is involved in regulation of NK cell activity presumably when an excess of IFN appears in body tissues and fluids.

It is interesting that the presence of the IIRA in IFN-containing supernatants, and its 'antiregulatory' action on the properties of IFNs do not change the antiviral activity of preparations, which can be evaluated by a standard method that tests the protective effect of IFN on monolayer human fibroblast cultures infected with VSV.[31] These supernatant preparations abolished the auto-IFN-induced stimulation of PBL possessing NK activity in MS patients up to the values of TRPs characteristic of the initial, untreated suspension of PBL. Thus, the blocking properties of the IIRA can be exerted in both an autologous and an allogeneic system.

MS represents a multifactorial immunological abnormality associated with a strong deficiency of NK activity[14–17] probably caused by a functional defect of the IFN system.[18,35] This IFN-dependent NK deficiency is linked with a reduction in IFN production by practically all types of cells involved in the process.[18,19,22] It is also associated with anomalous *in vitro* responses of NK themselves as PBL possessing NK activity to the IFN regulatory action,[14,17,20] and is probably located at the stage of NK differentiation controlled by IFN. An interleukin (IL)-dependent step of maturation may also be involved, because IL-2 was found to augment NK activity of PBL in MS patients more slowly than in healthy donors, and induced less IFN-γ.[36]

On the other hand, some authors describe activatory changes in MS patients. Maintenance of excess IFN in serum and cerebrospinal fluid of MS patients was reported by Degré *et al.*[24] and Hertzog *et al.*,[19] accompanied by dissociation between the cytotoxic and IFN-producing functions of PBL possessing NK activity.[23] Furthermore, Salazar *et al.*[37] review properties of serum and tissue IFN-γ that induces demyelination in MS, while IFNs produced in the central nervous system induce remission.

### References

1. Herberman RB, Ortaldo JR. Natural killer cells: their role in defenses against disease. *Science* 1981; **214**: 24–30.
2. Saksela E, Timonen T, Cantell K. Human natural killer cell activity is augmented by interferon via recruitment of 'pre-NK' cells. *Scand J Immunol* 1979; **10**: 257–266.
3. Targan S, Dorey F. Interferon activation of 'pre-spontaneous killer' (pre-SK) cells and alteration in kinetics of lysis of both 'pre-SK' and active SK cells. *J Immunol* 1980; **124**: 2157–2161.
4. Whiteside TL, Herberman RB. The role of natural killer cells in human disease. *Clin Immunol Immunopathol* 1989; **53**: 1–23.
5. Bergsagel DE, Haas RH, Messner HA. Interferon in the treatment of chronic granulocytic leukemia. In: Silver HKB, ed. *Interferons in Cancer Treatment.* Toronto: MES Inc, 1986, 59–66.
6. Drouin J. Hairy cell leukemia: the role of therapy with interferons. In: Silver HKB, ed. *Interferons in Cancer Treatment.* Toronto: MES Inc, 1986; 31–39.
7. Conte R, Dinota A, Tazzari PL, Belletti D, Sermasi G. Analysis of natural killer cells in patients with idiopathic autoimmune hemolytic anemia. *Vox Sang* 1989; **56**: 270–273.
8. Galvani DW, Owens W, Nethersell ABW, Cawley JC. The beneficial effects of α-IFN in CGL are probably not mediated by NK cells. *Brit J Haematol* 1989; **71**: 233–237.
9. Galvani DW, Nethersell ABW, Cawley JC. α-interferon in myelodysplasia: clinical observations and effects on NK cells. *Leukemia Res* 1988; **12**: 257–262.
10. Hartung HP, Heininger K. Non-specific mechanisms of inflammation and tissue damage in MS. *Res Immunol* 1989; **140**: 226–233.
11. Johnson KP. Treatment of multiple sclerosis with various interferons: The cons. *Neurology* 1988; **38(suppl.2)**: 62–64.
12. Knobler RL. Systemic interferon therapy in multiple sclerosis: The pros. *Neurology* 1988; **38(suppl.2)**: 58–61.
13. Panitch HS, Haley AS, Hirsch RL, Johnson KP. Exacerbations of multiple sclerosis in patients treated with gamma interferon. *Lancet* 1987; **1**: 893–895.
14. Neighbour PA, Grayzel AI, Miller AE. Endogenous and interferon-augmented natural killer cell activity of human peripheral blood mononuclear cells *in vitro.* Studies of patients with multiple sclerosis, systemic lupus erythematosus or rheumatoid arthritis. *Clin Exp Immunol* 1982; **49**: 11–21.
15. Sorikin AM, Koval'chuk LV, Tunkel OI, Cheknev SB. Interferon-producing and cytotoxic activities of natural killer cells in patients with multiple sclerosis. *Zh Nevrol Psikh Imeni SS Korsakova* 1987; **87**: 211–215.
16. Stöger I, Tálas M, Bengzúr M, *et al.* Lack of correlation between interferon production and natural killer activity of lymphocytes in multiple sclerosis. *Arch Virol* 1982; **71**: 259–265.
17. Uchida A, Maida EM, Lenzhofer R, Micksche M. Natural killer cell activity in patients with multiple sclerosis: interferon and plasmapheresis. *Immunobiol* 1982: **160**: 392–402.
18. Cheknev SB, Latysheva OL, Tunkel OI, *et al.* Interferon status in multiple sclerosis. *Immunologiya* 1990; **N6**: 57–60 (in Russian).
19. Hertzog PJ, Wright A, Harris G, Linnane AW, Mackay IR. Intermittent interferonemia and interferon responses in multiple sclerosis. *Clin Immunol Immunopathol* 1991; **58**: 18–32.
20. Cheknev SB, Sorokin AM, Tunkel OI. Individual response of human natural killer cells to interferon regulatory effects *in vitro. Immunologiya* 1990; **N6**: 47–50 (in Russian).
21. Kasahara T, Djeu JY, Dougherty SF, Oppenheim JJ. Capacity of human large granular lymphocytes (LGL) to produce multiple lymphokines: interleukin 2, interferon, and colony stimulating factor. *J Immunol* 1983; **131**: 2379–2389.
22. Sorokin AM, Cheknev SB, Koval'chuk LV. The role of adherent cells in ensuring reactions of natural cytotoxicity. *Byull Eksper Biol Med* 1989; **108**: 322–325.
23. Cheknev SB, Sorokin AM, Koval'chuk LV. Dissociation in the cytotoxic and interferon-producing activities of natural killer cells in the pathogenesis of immunoregulatory imbalance. *Immunologiya* 1989; **N3**: 48–52 (in Russian).
24. Degré M, Dahl H, Vandvik B. Interferon in the serum and cerebrospinal fluid in patients with multiple sclerosis and other neurological disorders. *Acta Neurol Scand* 1976: **53**: 152–160.
25. McDonald WJ, Halliday BM. Diagnosis and classification of multiple sclerosis. *Brit Med Bull* 1977; **33**: 4–9.
26. Böyum A. Isolation of mononuclear cells and granulocytes from human blood. *Scand J Clin Lab Invest* 1968; **21(suppl.97)**: 77–89.
27. Hashimoto Y, Sudo H. Evaluation of cell damage in immune reactions by release of radioactivity from 3H-uridine labelled cells. *GANN* 1971; **62**: 139–143.
28. Rykova MP, Spirande IV, Zedgenidze MS, Lyakhov VV, Fuks BB. New high sensitive technique for testing natural killers. *Immunologiya* 1981; **N3**: 88–90 (in Russian).
29. Kirchner H, Kleinicke CH, Digel W. A whole blood technique for testing production of human interferon by leukocytes. *J Immunol Meth* 1982; **48**: 213–219.
30. Grigoryan SS, Maiorov IA, Ivanova AM, Yershov FI. Evaluation of interferon status of donors in probes of whole blood. *Vopr Virusol* 1988; **N4**: 433–436.
31. Solov'ev VD, Bektemirov TA. Leukocyte interferon as a parameter of reactivity of organism in normal and pathological conditions. *Vestn Akad Med Nauk SSSR* 1979; **N2**: 19–26 (in Russian).
32. Fleishmann WR, Georgiades JA, Osborne LC, Dianzani F, Johnson HM. Induction of an inhibitor of interferon action in a mouse lymphokine preparation. *Infect Immun* 1979; **26**: 949–955.
33. Marchenko VI, Pokidisheva LN, Orlova NG. Cryomethod for concentrating interferon. *Vopr Virusol* 1975; **N5**: 568–570.
34. Cheknev SB, Narovlyanskii AN, Amchenkova AM, Ershov FI. Regulation of human natural killer cell activity by an interferon inhibitor. *Byull Eksper Biol Med* 1991; **112**: 1454–1456.

35. Cheknev SB, Koval'chuk LV. Regulation of human natural killer cell activity. In: Koval'chuk LV, Cheredeev AN, eds. *Mechanisms of Immunoregulation and Immune Biotechnology.* Moscow: 2nd Medical Institute, 1989; 12–16 (in Russian).
36. Braakman E, van Tunen A, Meager A, Lucas CJ. Natural cytotoxic activity in multiple sclerosis patients: defects in IL-2/interferon γ-regulatory circuit. *Clin Exp Immunol* 1986; **66**: 285–294.
37. Salazar AM, Gibbs CJ Jr, Gajdusek DC, Smith RA. Clinical use of interferons: central nervous system disorders. In: Came PE, Carter WA, eds. *Handbook of Experimental Pharmacology Vol 71.* Berlin, Heidelberg: Springer-Verlag, 1984; 471–497.

**Received 10 February 1994; accepted in revised form 25 May 1994**
